# Head, face, and neck injury patterns across the life course: a 20-year NEISS analysis of 2,345,287 emergency department visits in the U.S.

**DOI:** 10.1186/s12873-026-01821-3

**Published:** 2026-10-20

**Authors:** Michael Alfertshofer, Cosima C. Hoch, Dirk Nolte, Hans-Günther Machens, Leonard Knoedler, Shady Abu-Sirhan, Siti Rahmayanti, Deise Caldas Kühlman, Jeffrey E. Janis, Zachary S. Peacock, Steffen Koerdt, Sarina Bucher, Max Heiland, Nicholas Moellhoff, Samuel Knoedler

**Affiliations:** 1 Department of Plastic Surgery and Hand Surgery, Technical University of Munich, Munich, Germany; 2 Department of Oral and Maxillofacial Surgery, Charité – Universitätsmedizin Berlin, Corporate Member of Freie Universität Berlin and Humboldt-Universität zu Berlin, Augustenburger Platz 1, 13353 Berlin, Germany; 3 Department of Otorhinolaryngology, Head and Neck Surgery, University Hospital Zurich, University of Zurich, Zurich, Switzerland; 4 Department of Oral and Maxillofacial Surgery, Ludwig-Maximilians-Universität München, Munich, Germany; 5 Division of Plastic Surgery, Department of Surgery, Brigham and Women’s Hospital, Boston, MA USA; 6 Department of Preventive and Community Dentistry, Faculty of Dental Surgery, Rio de Janeiro State University, Rio de Janeiro, Brazil; 7 Department of Oral and Maxillofacial Surgery, Amsterdam UMC, University of Amsterdam, Amsterdam, the Netherlands; 8 Department of Plastic and Reconstructive Surgery, College of Medicine, The Ohio State University Wexner Medical Center, Columbus, OH USA; 9 Department of Oral and Maxillofacial Surgery, Harvard School of Dental Medicine, Harvard University, Boston, MA USA; 10 Cleft and Craniofacial Clinic, Mass General Hospital for Children and Shriners Hospital for Children, Boston, MA USA; 11 Division of Hand, Plastic and Aesthetic Surgery, Ludwig-Maximilians-University Munich, Munich, Germany

**Keywords:** Facial injuries, National Electronic Injury Surveillance System, Emergency service, hospital, Accidental falls, Maxillofacial injuries

## Abstract

**Background:**

Head, face, and neck injuries impose a substantial burden for emergency departments (ED); the mechanisms driving them across the life course however remain poorly understood at the population level. Large-scale, integrated analysis spanning anatomical regions, injury mechanisms, age groups, settings, and associated products or environmental surfaces offers an opportunity to identify the patterns and vulnerable populations that smaller or shorter-term studies cannot resolve.

**Methods:**

We queried National Electronic Injury Surveillance System (NEISS) records from 2006 to 2025 for injuries to the head, face, eyes, mouth, neck, or ears. Demographics, anatomical region, injury setting, associated products or environmental surfaces, and ED disposition were extracted, and a rule-based narrative classifier assigned 1 of 17 injury mechanisms from free-text descriptions. Non-routine ED disposition was defined as admission, transfer, observation, or fatality. Analyses were descriptive and based on unweighted NEISS case records.

**Results:**

Among 2,345,287 ED presentations, representing 31.8% of all NEISS-coded visits, the head was most often injured (51.2%), followed by the face (28.2%). Annual volume rose 35.2% (105,727 in 2006 to 142,918 in 2025), driven mainly by head injuries (+ 83.4%). Median age was 14 years and 56.4% of patients were male, but among older adults (≥ 65 years), females predominated (60.6%). Falls were the leading mechanism (53.0% across subcategories). Most patients were treated and discharged (89.4%) and admission rate rose roughly 18-fold from toddlers (1.3%) to older adults (23.3%). Non-routine ED disposition increased with age, and syncope- or medical-related falls had the highest non-routine ED disposition rate (33.9%). The home was the most common injury setting (63.1%). Injury exposures varied by age group, with beds most common in infants, tables in toddlers, sports equipment in children and adolescents, and floors in older adults.

**Conclusions:**

Head, face, and neck injuries represent a large and growing share of NEISS-coded ED presentations, with distinct patterns by age, sex, injury mechanism, setting, and clinical outcome. These findings may assist in the planning of targeted prevention strategies and future research on risk stratification for vulnerable age groups and injury mechanisms.

## Background

Injuries to the head, face, and neck comprise a heterogeneous group of emergency department (ED) presentations, ranging from lacerations, contusions, retained foreign bodies to fractures, burns, dental trauma, ocular injuries, and cervical trauma. Collectively, these injuries represent a substantial clinical and public health burden in the United States [1, 2]. Prior national estimates suggest that more than 5.4 million ED visits annually are attributable to primary head and neck injuries, with open wounds being the most common diagnosis and laceration repair the most frequently performed procedure [1]. Facial fractures alone account for approximately 480,000 ED visits per year in the United States, with associated ED charges approaching $1 billion annually, while ocular injuries contribute an additional 700,000 to 2 million ED visits each year [3–5]. Beyond the immediate clinical workload, the broader economic burden of injury remains considerable, with total injury-related costs in the United States estimated at $4.2 trillion in 2019, including $327 billion in direct medical care costs [2]. 

The epidemiology of head, face, and neck injuries is shaped by age, sex, injury mechanism, anatomical region, environmental context, and consumer product exposure. Falls are consistently identified as one of the leading mechanisms of craniofacial trauma across the lifespan, particularly among young children and older adults [1, 6, 7]. In the United States, recent analyses have suggested an increasing burden of facial fractures, with rising mean patient age and a growing proportion of female patients [8]. Similar patterns have been observed in aging populations, where fall-related hospitalizations and facial fracture hospitalization rates have increased disproportionately over time [9, 10]. These trends likely reflect the combined effects of population aging, changing activity patterns, evolving consumer product exposures, and shifts in ED utilization and clinical evaluation practices [11, 12]. 

Despite the recognized importance of these injuries, several gaps remain in the literature. Many epidemiological studies have focused on isolated anatomical subregions, such as the face, globe, ear or teeth rather than the entire head and neck [4, 8, 13, 14]. Other studies have examined head and neck injuries more broadly, but have often been limited to single-year or short-term observation periods, restricting the ability to characterize long-term trends [1, 15]. Furthermore, few large-scale studies have simultaneously examined demographic distributions, anatomical injury patterns, injury mechanisms, consumer products, injury settings, and ED outcomes across the full life course [16, 17]. This integrated perspective is important because mechanisms and risk environments differ substantially between infants, children, adolescents, working-age adults, and older adults, and prevention strategies must therefore be tailored to age-specific exposures and vulnerabilities.

The National Electronic Injury Surveillance System (NEISS), maintained by the U.S. Consumer Product Safety Commission, provides a unique opportunity to address these limitations. NEISS captures standardized data from a stratified probability sample of approximately 100 U.S. hospital EDs and includes information on patient demographics, anatomical body part, diagnosis, ED disposition, injury location, implicated consumer products, and brief free-text narratives describing the circumstances of injury [18, 19]. Although NEISS does not contain a structured injury-mechanism variable, the narrative field allows mechanism-level information to be derived through systematic text classification.

The objectives of this study were therefore to: (1) characterize the epidemiology of head, face, and neck injuries presenting to NEISS-participating EDs over a 20-year period from 2006 to 2025, including temporal trends, demographic distributions, anatomical patterns, and associated products or environmental surfaces; (2) develop and validate an automated injury-mechanism classifier to enable mechanism-level analysis from NEISS free-text narratives; (3) describe the distribution of injury mechanisms and associated products or environmental surfaces across age groups and anatomical regions; and (4) evaluate clinical outcomes, including admission, transfer, observation, fatality, and non-routine ED disposition rates, by age, sex, anatomical region, and mechanism. By providing an integrated age-stratified analysis of these injuries, this study aims to identify vulnerable populations, clarify mechanism- and product-specific risk patterns, and inform targeted prevention strategies.

## Materials and methods

### Data source

This study is a retrospective analysis of de-identified case records from the U.S. Consumer Product Safety Commission (CPSC) National Electronic Injury Surveillance System (NEISS) for the calendar years 2006 through 2025. NEISS is a stratified probability sample of approximately 100 U.S. hospital EDs, from which trained abstractors code every consumer-product-related injury treated in the sampled EDs onto a standardized case record. The sampling frame of NEISS includes hospitals in the U.S. with a minimum of six beds and a 24-hour ED, excluding psychiatric and penal institutions. The sampling frame is divided into five strata: four strata defined by annual ED visit volume (i.e., small, medium, large, and very large) and a fifth stratum of children’s hospitals. Hospitals are selected depending on probability sampling within each stratum, and each case record carries a statistical weight based on the selection probability of the respective hospital. The geographic location and hospital-level details of the participating institutions such as trauma center designation are not disclosed in the public-use data [20]. Each record contains a date of treatment, single-year patient age, sex, race/ethnicity, a primary anatomical body part and ICD-coded diagnosis, the disposition at the conclusion of the ED visit, the patient’s location at the time of injury, up to three associated consumer products (CPSC product codes), indicator variables for fire, alcohol, and drug involvement, a brief free-text narrative summarizing the circumstances of injury, and the sampling stratum, primary sampling unit (PSU), and statistical weight assigned by CPSC [21]. Product codes were interpreted as associated products or environmental surfaces recorded in NEISS and do not necessarily imply product causality. The NEISS public-use data are de-identified and exempt from human-subjects review; hence this study did not require institutional review board (IRB) approval. Regardless, IRB approval was obtained prior to study commencement from the ethics committee of the Technical University of Munich under approval number: 2024-80-S-CB.

### Cohort definition

**Fig. 1:**
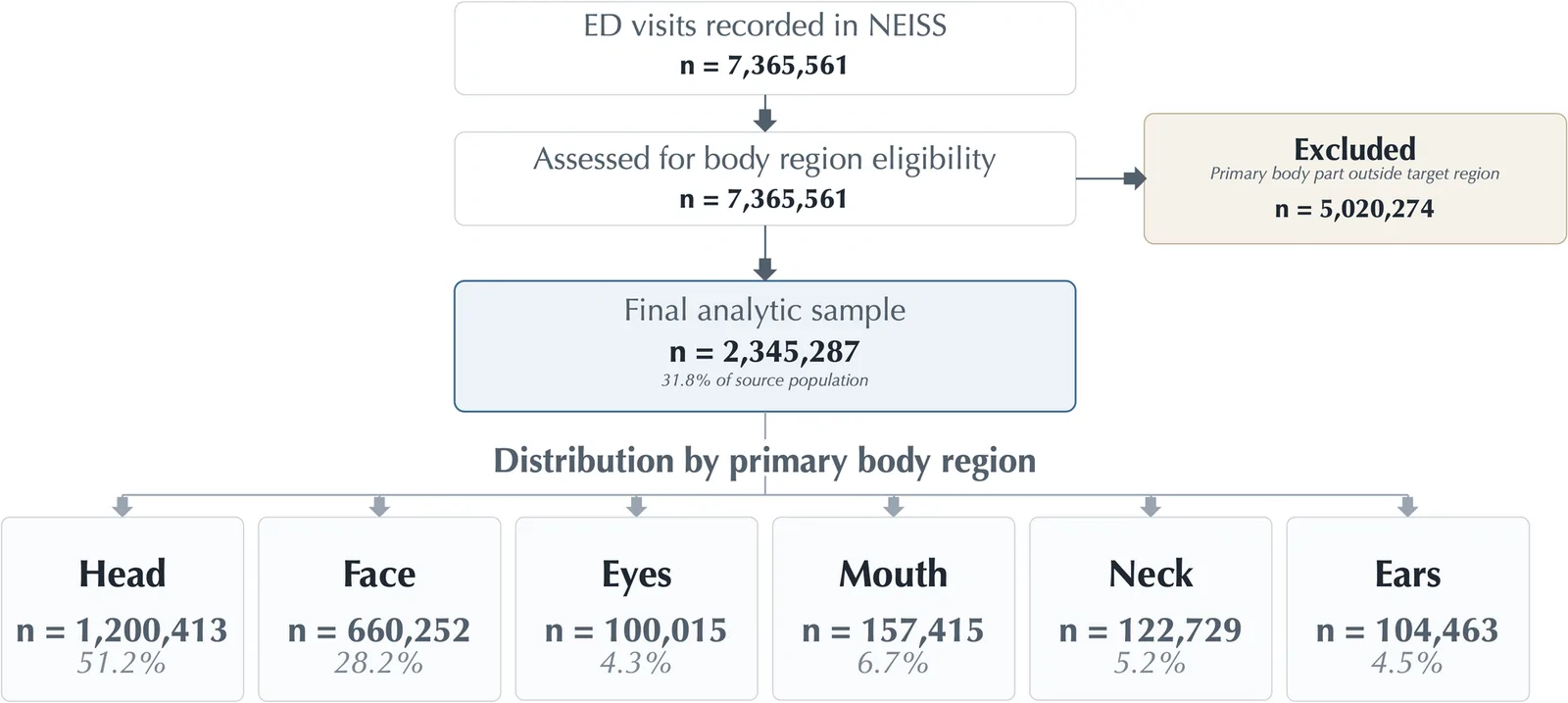
Flow diagram of cohort selection and distribution by primary body region. Of 7,365,561 NEISS-coded ED visits recorded between 2006 and 2025, 2,345,287 visits involved one of the target head, face, or neck regions and were included in the final analytic sample. The lower panel shows the distribution of included cases by primary body region: head, face, eyes, mouth, neck, and ears. Counts are unweighted NEISS case records

Eligible cases included all NEISS records from 2006 to 2025 in which the primary body part field corresponded to head (code = 75), face (code = 76), eyeball (code = 77), mouth (code = 88), neck (code = 89), or ear (code = 94) injuries. Because cohort selection was based on the primary coded body part rather than detailed imaging or diagnostic granularity, the dataset did not allow reliable separation of extracranial from intracranial head injuries. Records were excluded if the primary body part field corresponded to any anatomical region outside these six target regions. No additional exclusion criteria were applied. The mapping of NEISS Body Part codes to anatomical labels was verified against the CPSC NEISS Coding Manual and independently cross-checked against a random sample of 500 case narratives per code to confirm anatomical validity. The final study cohort comprised 2,345,287 ED presentations across the 20-year window (Fig. 1).

**Fig. 2:**
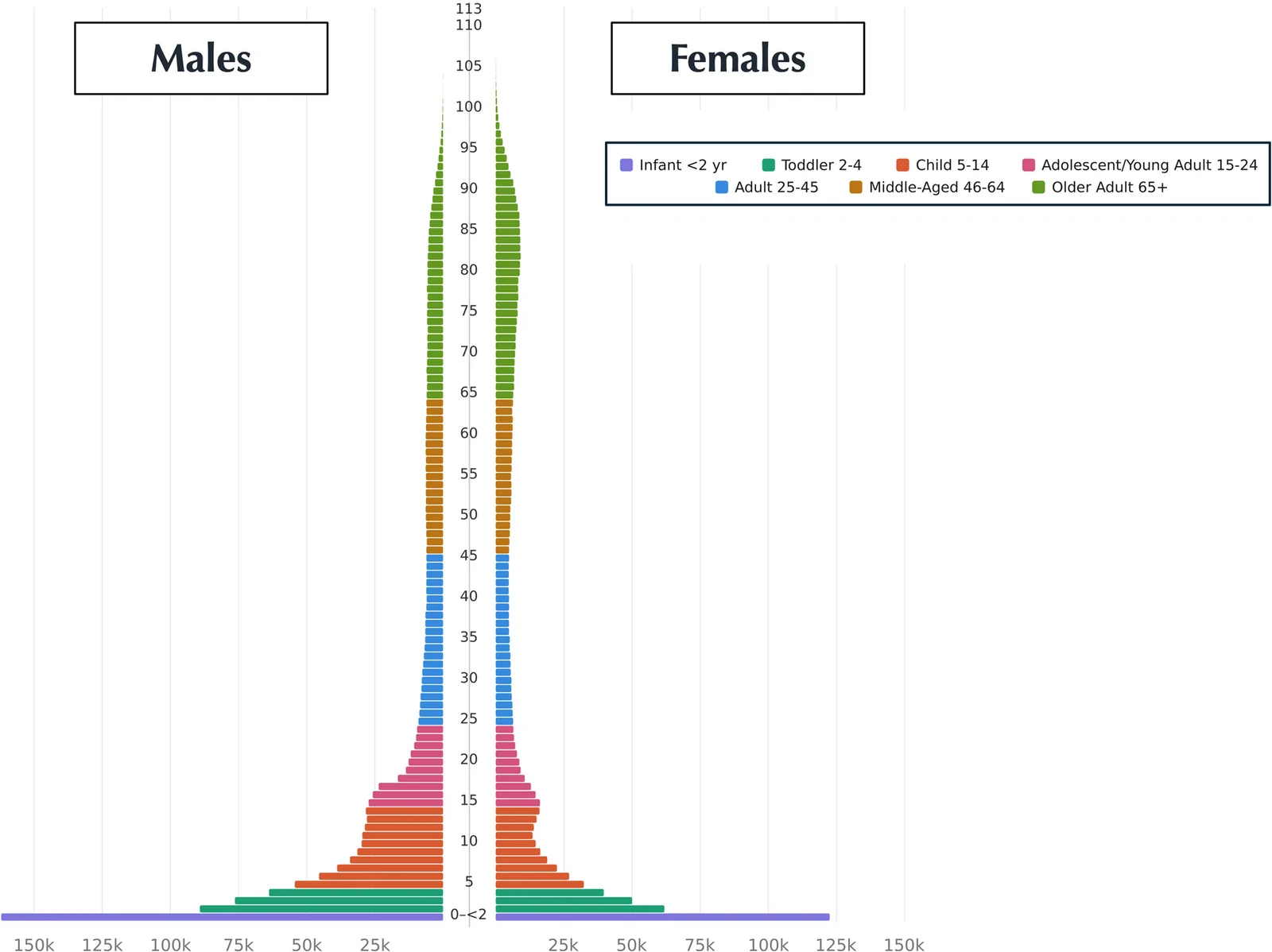
Age- and sex-specific distribution of included head, face, and neck injury presentations. The population-pyramid plot shows case counts by single-year age, with males displayed on the left and females on the right. Colors indicate the predefined age groups used in the analysis. Counts are unweighted NEISS case records

Patient sex was recoded from the NEISS native values to male, female, or unknown; sex was unrecorded for 321 records (0.01%). To allow consistent grouping across the lifespan, every patient record was assigned to one of seven mutually exclusive age categories: *Infant* (< 2 years), *Toddler* (2–4 years), *Child* (5–14 years), *Adolescent / Young Adult* (15–24 years), *Adult* (25–45 years), *Middle-Aged Adult* (46–64 years), and *Older Adult* (≥ 65 years). Records with unknown age (< 0.01%) were retained for region-level totals but excluded from any analysis that required an age group (Fig. 2).

ED disposition was retained at the native NEISS coding (1 = treated and released; 2 = treated and transferred to another hospital; 4 = treated and admitted/hospitalized at the index hospital; 5 = held for observation; 6 = left without being seen or against medical advice; 8 = fatality, including patients dead on arrival or in the ED; 9 = not recorded). Injury location was retained at the native NEISS coding (0 = not recorded; 1 = home; 2 = farm or ranch; 4 = street or highway; 5 = other public property; 6 = mobile or manufactured home; 7 = industrial; 8 = school or daycare; 9 = place of recreation or sports). Consumer products were retained at the native CPSC three- or four-digit product-code level (*Product_1*, the primary product associated with the injury); the top ten product codes by frequency were extracted within each age group for the life-course product analysis.

### Injury mechanism classification

NEISS does not include a structured and standardized injury-mechanism variable; the circumstances of each injury are documented only in a brief, abbreviation-laden free-text narrative (median length 83 characters) captured by the abstractor. To enable mechanism-level analysis, we developed an automated rule-based narrative classifier that assigned each case to one of 17 mutually exclusive injury-mechanism categories or to a residual “Other/Unspecified” category. The 17 mechanism categories were defined a priori on the basis of standard injury-epidemiology taxonomies and the Centers for Disease Control and Prevention’s ICD-10-based external-cause-of-injury matrix, and comprised: (1) *Fall-same level* (2) Fall-from height (i.e., any fall from an elevated surface, including beds, furniture, and stairs) (3) *Fall-syncope/medical* (4) *Sports & recreation* (5) *Cycling & wheeled (non-motor)* (6) *Motor vehicle related* (7) *Struck against object* (8) *Struck by object* (9) *Crush/caught* (10) *Cut/pierce by sharp object* (11) *Burn/thermal/electrical* (12) *Foreign body-inserted or aspirated* (13) *Overexertion/muscle strain* (14) *Substance-related* (15) *Assault/interpersonal violence* (16) *Animal/insect-related* and (17) *Other/Unspecified* [22, 23]. 

### Classifier development, refinement and validation

The classifier was implemented as an ordered list of regular-expression rules applied to the narrative text. Each rule consisted of one or more regular-expression patterns matching narrative tokens, phrases, NEISS abbreviation conventions, and common typographical variants known to occur in the data (e.g., “TD&F” for “tripped, down and fell”; “FB” for “foreign body”; “ETOH” for ethanol; “LAC” for laceration; “MVA” for motor vehicle accident). Rules were tested in priority order and the first matching rule determined the assigned category, so that more specific mechanisms (e.g., assault, substance use, motor vehicle, cycling, and sports) were evaluated before generic fall and struck-against patterns. The classifier was developed iteratively across four refinement cycles: each cycle inspected a stratified random sample of cases assigned to the residual “Other/Unspecified” category, identified recurring narrative patterns that ought to have been classifiable, and extended or tightened the rule set accordingly. The final ruleset contained more than 200 distinct surface tokens (objects, surfaces, sports, mechanisms, and anatomical references) and approximately 18 ordered rule blocks. After the fourth refinement cycle, the proportion of cases falling into the residual “Other/Unspecified” category had been reduced from 20.3% (initial classifier) to 5.9% (final classifier) on the full 20-year cohort. Two validation procedures were performed. First, a fixed sanity-test panel of 54 hand-curated narrative-category pairs (designed to span all 17 categories and to include deliberate edge cases such as drink-related falls, sports-related foreign bodies, and head-to-head collisions during play) was used as a regression test after every refinement cycle; the final classifier achieved 100% agreement on this panel. Second, the classifier was applied independently to five 4-year sub-blocks of the dataset and to the full pooled cohort, yielding consistent category proportions across blocks.

### Statistical analysis

Data assembly and analytic computations were performed in Python 3.12 (standard library with the openpyxl, pandas, NumPy, and Matplotlib packages); cross-tabulations were additionally reproduced in IBM SPSS Statistics 29. Analyses were descriptive in nature. Continuous variables (patient age in years) are reported as counts within categorical strata; categorical variables (sex, body region, age group, injury mechanism, ED disposition, injury location, and consumer product code) are reported as absolute frequencies and proportions. Proportions are expressed as a percentage of the column marginal, the row marginal, or the grand total, depending on the analytic question. NEISS sampling weights, strata, and primary sampling units were retained in the analytic dataset; all analyses reported here use unweighted case records, and counts and proportions therefore refer to the observed NEISS sample. An unweighted approach was chosen because the study aimed to describe relative injury patterns among captured cases rather than to estimate national incidence, and because weighted estimates for the many sparsely populated age, sex, region, and mechanism subgroups would have been statistically unstable. No imputation was performed.

## Results

### Patient demographic data

**Fig. 3:**
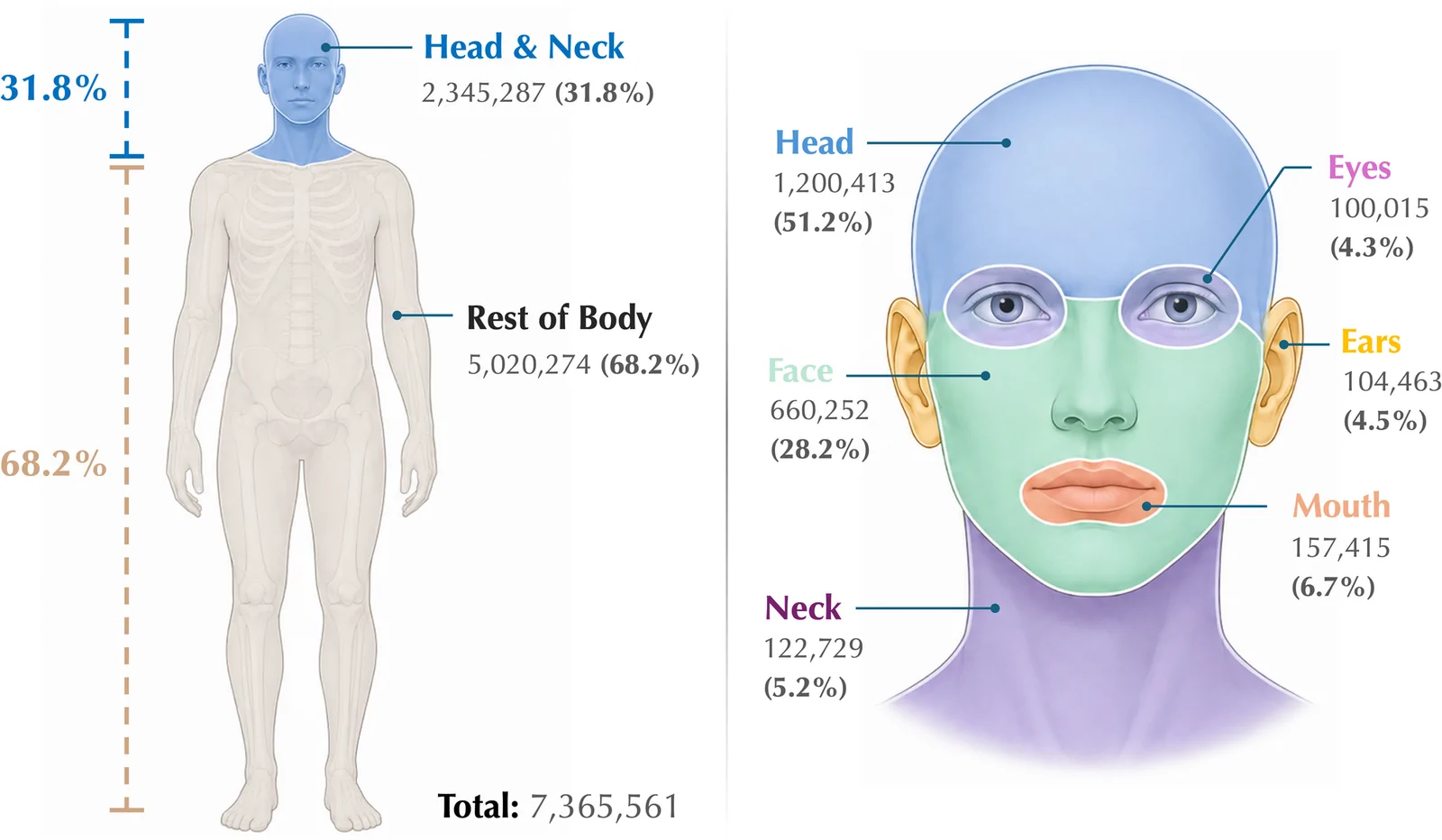
Anatomical distribution of target-region injuries among all NEISS-coded ED visits. The left panel illustrates the proportion of all NEISS-coded visits involving the target head, face, and neck regions compared with the rest of the body. The right panel shows the distribution of injuries across the six included anatomical regions: head, face, eyes, mouth, neck, and ears. Counts and percentages are based on unweighted NEISS case records

Over the 20-year study period, 2,345,287 ED visits for injuries to the head, face, neck, mouth, eyes, and ears were captured, accounting for 31.8% of all NEISS-coded ED visits (Fig. 3). Patients spanned ages 0 to 113 years, with a median age of 14 years [IQR 4–49] and a mean age of 27.1 ± 28.7 years. Male patients accounted for 56.4% (1,322,876) and female patients for 43.6% (1,022,090) of cases; sex was not recorded for 321 patients (0.01%).

The head was the most frequently injured region (51.2%; 54.1% male), followed by the face (28.2%; 59.6% male), mouth (6.7%; 62.1% male), neck (5.2%; 51.2% male), ears (4.5%; 50.4% male), and eyes (4.3%; 67.1% male) (Table 1). The sex distribution varied across age groups, ranging from a male predominance among Children (64.7% male vs. 35.3% female) to a female predominance among Older Adults (60.6% female vs. 39.4% male), the only age group in which female cases exceeded male cases (Table 2).

**Table 1:** Injured body region by patient age group, NEISS 2006–2025

Age group	Age range	Head	Face	Eyes	Mouth	Neck	Ears	Row total
Infant	< 2 yr	167,606 58.9%	77,598 27.3%	4,662 1.6%	29,560 10.4%	1,088 0.4%	4,068 1.4%	**284**,**582** 100%
Toddler	2–4	147,766 38.8%	154,900 40.7%	9,202 2.4%	45,572 12.0%	5,019 1.3%	18,427 4.8%	**380**,**886** 100%
Child	5–14	245,605 45.6%	156,719 29.1%	21,912 4.1%	42,600 7.9%	25,937 4.8%	46,156 8.6%	**538**,**929** 100%
Adolescent/Young Adult	15–24	128,628 49.1%	69,810 26.7%	14,705 5.6%	16,513 6.3%	18,142 6.9%	14,101 5.4%	**261**,**899** 100%
Adult	25–45	114,198 44.1%	64,739 25.0%	26,996 10.4%	12,187 4.7%	28,773 11.1%	11,833 4.6%	**258**,**726** 100%
Middle-Aged Adult	46–64	125,280 55.1%	51,604 22.7%	16,439 7.2%	6,054 2.7%	22,038 9.7%	5,936 2.6%	**227**,**351** 100%
Older Adult	65+	271,190 69.1%	84,831 21.6%	6,097 1.6%	4,928 1.3%	21,721 5.5%	3,941 1.0%	**392**,**708** 100%
Column total		**1**,**200**,**273** 51.2%	**660**,**201** 28.2%	**100**,**013** 4.3%	**157**,**414** 6.7%	**122**,**718** 5.2%	**104**,**462** 4.5%	**2**,**345**,**081**

Cells show case counts with the region’s percentage within each age group below (rows sum to 100%). Unweighted case counts; 206 unknown-age records excluded

**Table 2:** Injured body region by patient age group and sex, NEISS 2006–2025

Age group	Head		Face		Eyes		Mouth		Neck		Ears	
	Male	Female	Male	Female	Male	Female	Male	Female	Male	Female	Male	Female
Infant (< 2 year)	92,587 57.1%	74,999 61.2%	46,662 28.8%	30,931 25.3%	2,595 1.6%	2,066 1.7%	17,861 11.0%	11,696 9.5%	617 0.4%	471 0.4%	1,739 1.1%	2,329 1.9%
Toddler (2–4 year)	91,410 39.9%	56,346 37.2%	92,581 40.4%	62,309 41.1%	5,469 2.4%	3,733 2.5%	27,936 12.2%	17,634 11.6%	2,883 1.3%	2,135 1.4%	9,079 4.0%	9,346 6.2%
Child (5–14 year)	165,480 47.5%	80,100 42.1%	102,538 29.4%	54,166 28.5%	14,168 4.1%	7,743 4.1%	27,946 8.0%	14,646 7.7%	15,821 4.5%	10,112 5.3%	22,778 6.5%	23,373 12.3%
Adolescent / Young Adult (15–24 year)	74,400 46.1%	54,183 54.0%	48,650 30.1%	21,150 21.1%	10,574 6.6%	4,131 4.1%	11,264 7.0%	5,243 5.2%	9,659 6.0%	8,477 8.4%	6,880 4.3%	7,219 7.2%
Adult (25–45 year)	59,491 40.3%	54,666 49.2%	40,888 27.7%	23,837 21.4%	19,412 13.2%	7,578 6.8%	7,493 5.1%	4,690 4.2%	13,903 9.4%	14,864 13.4%	6,325 4.3%	5,506 5.0%
Middle-Aged Adult (46–64 year)	61,255 51.6%	64,005 59.0%	28,640 24.1%	22,957 21.1%	11,239 9.5%	5,197 4.8%	3,245 2.7%	2,809 2.6%	10,929 9.2%	11,104 10.2%	3,439 2.9%	2,495 2.3%
Older Adult (≥ 65 year)	104,792 67.7%	166,384 70.0%	33,143 21.4%	51,682 21.7%	3,680 2.4%	2,417 1.0%	1,936 1.2%	2,991 1.3%	8,960 5.8%	12,760 5.4%	2,378 1.5%	1,563 0.7%
Total	649,415 49.1%	550,683 53.9%	393,102 29.7%	267,032 26.1%	67,137 5.1%	32,865 3.2%	97,681 7.4%	59,709 5.8%	62,772 4.7%	59,923 5.9%	52,618 4.0%	51,831 5.1%

Each region is split into male and female sub-columns. Cells show case counts with the percentage within each age-sex stratum below; percentages sum to 100% across body regions within each age-sex row. Unweighted case counts; records with unknown age and/or unknown sex were excluded

### Temporal trends

**Fig. 4:**
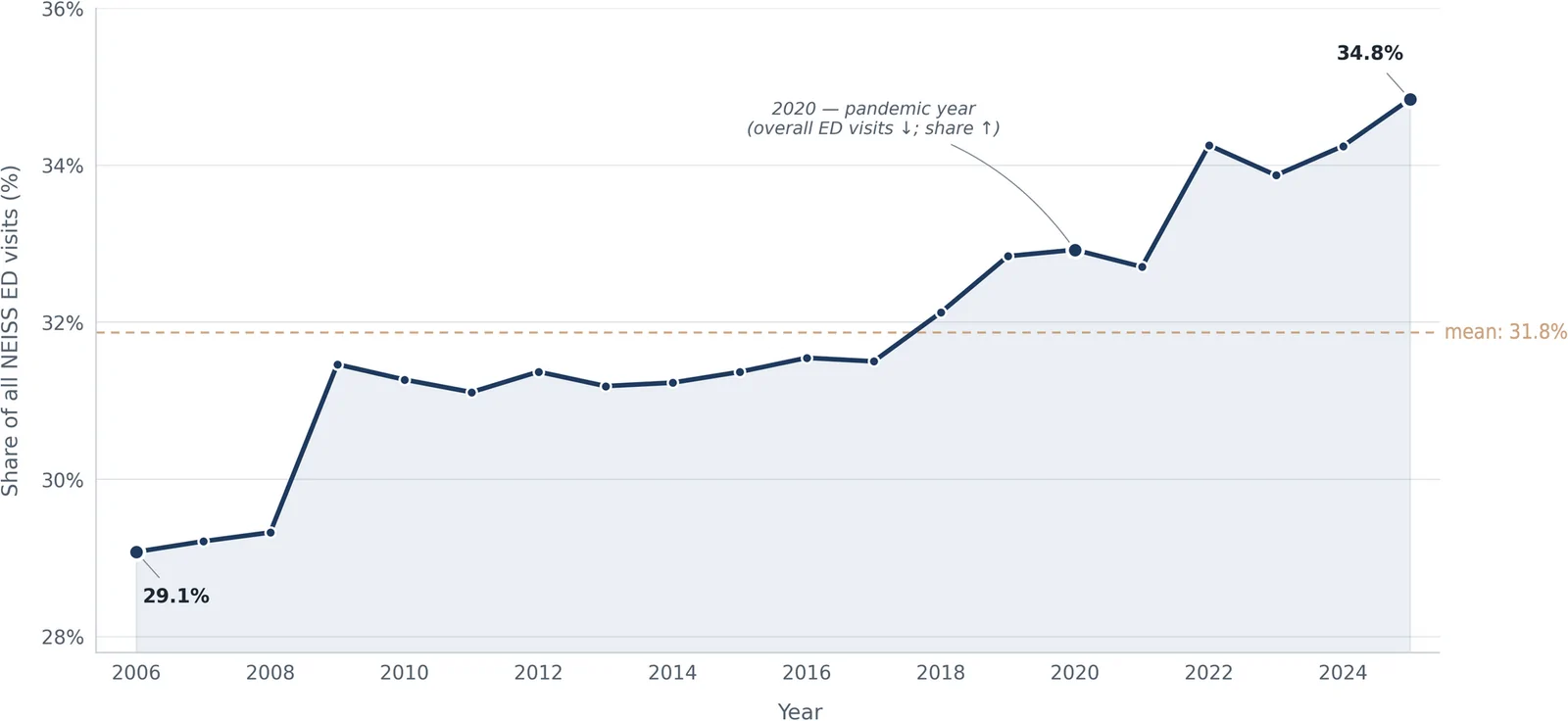
Temporal trend in the proportion of all NEISS-coded ED visits attributable to target head, face, and neck region injuries from 2006 to 2025. The line represents the annual share of all NEISS-coded ED visits involving the six target anatomical regions. The dashed horizontal line indicates the mean share across the study period. The year 2020 is annotated to indicate the COVID-19 pandemic period, during which overall ED utilization decreased

**Fig. 5:**
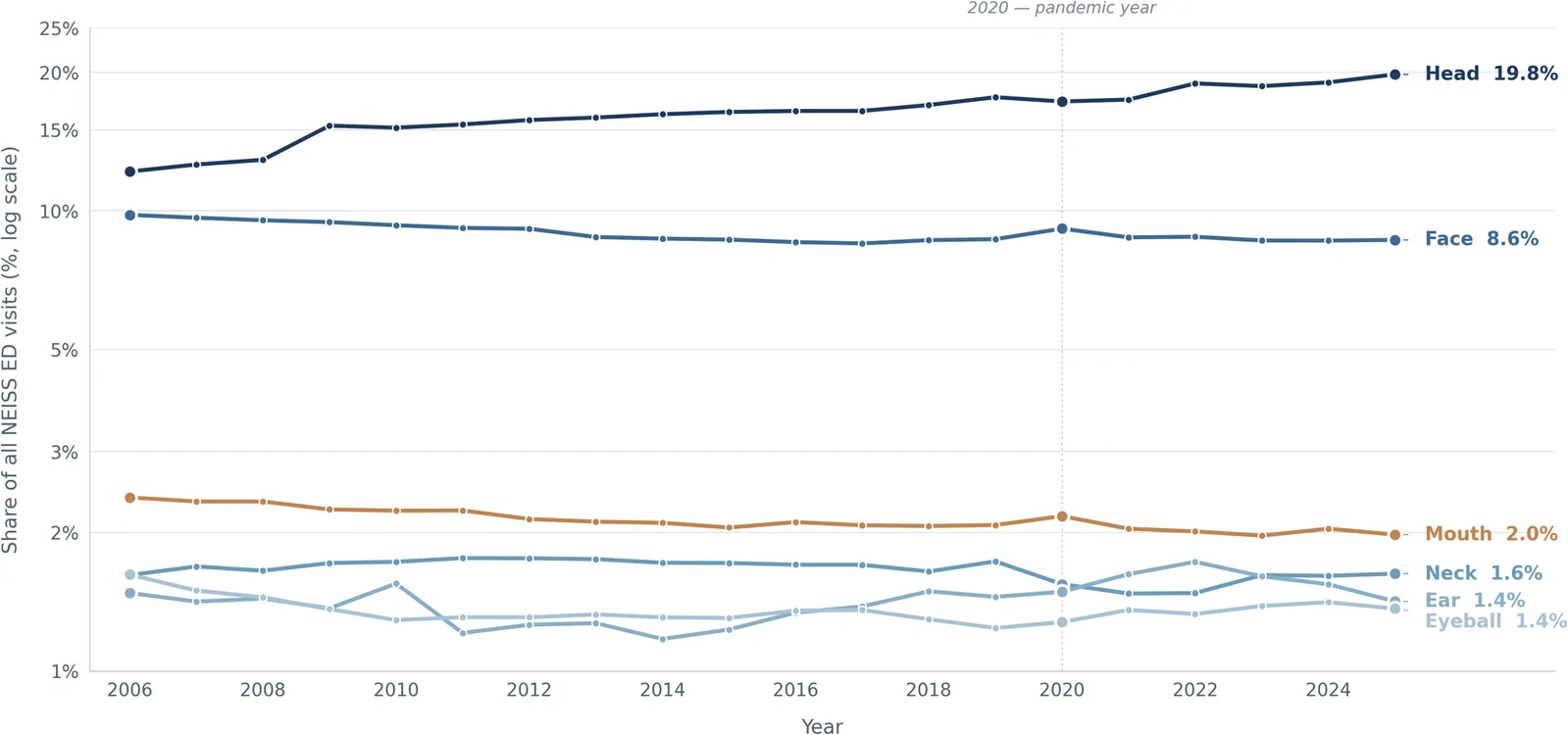
Temporal trends in target-region injuries by anatomical body region from 2006 to 2025. Lines show the annual share of all NEISS-coded ED visits attributable to each anatomical region. The y-axis is displayed on a logarithmic scale to allow visualization of both high-frequency regions, such as head and face, and lower-frequency regions, such as eyes, ears, mouth, and neck. Counts are unweighted NEISS case records

Annual case counts ranged from 101,854 in 2020 to 142,918 in 2025 (median 116,868 per year), corresponding to an increase of 35.2% between 2006 (105,727 cases) and 2025. The share of all NEISS-coded ED visits attributable to the target regions rose from 29.1% to 34.8%, an absolute increase of 5.7 percentage points (Table 3 and Fig. 4). Case volume in 2020 was the lowest of the study period, 13.5% below that of 2019, with reductions observed in every region. The overall increase was driven primarily by head injuries, with annual counts rising from 44,277 to 81,223 (+ 83.4%), whereas annual counts for face, eye, and mouth injuries remained essentially unchanged and those for neck (+ 13.6%) and ear (+ 8.2%) injuries rose moderately (Fig. 5).

**Table 3:** Emergency department visits for target head, face, and neck region injuries by year, NEISS 2006–2025

Year	Head	Face	Eyes	Mouth	Neck	Ears	Total	Share of all ED visits
2006	44,277	35,635	5,891	8,669	5,879	5,376	105,727	29.1%
2007	46,641	35,740	5,533	8,642	6,242	5,236	108,034	29.2%
2008	48,325	35,707	5,413	8,742	6,184	5,380	109,751	29.3%
2009	60,033	37,052	5,343	8,799	6,731	5,367	123,325	31.5%
2010	61,521	37,757	5,237	9,054	7,009	6,287	126,865	31.3%
2011	61,109	36,417	5,193	8,856	6,979	4,798	123,352	31.1%
2012	62,144	36,066	5,161	8,440	6,935	4,974	123,720	31.4%
2013	60,130	33,071	5,004	7,959	6,599	4,791	117,554	31.2%
2014	59,618	31,990	4,810	7,718	6,319	4,316	114,771	31.2%
2015	58,912	31,104	4,685	7,363	6,166	4,422	112,652	31.4%
2016	61,873	32,090	5,077	7,908	6,390	5,026	118,364	31.6%
2017	63,798	32,879	5,252	8,024	6,584	5,347	121,884	31.5%
2018	61,436	31,240	4,688	7,475	5,953	5,391	116,183	32.1%
2019	63,352	31,143	4,450	7,448	6,212	5,202	117,807	32.8%
2020	53,489	28,313	3,954	6,719	4,779	4,600	101,854	32.9%
2021	59,411	29,823	4,623	6,939	5,019	5,529	111,344	32.7%
2022	61,180	28,408	4,303	6,504	4,778	5,586	110,759	34.3%
2023	63,152	29,170	4,686	6,662	5,467	5,436	114,573	33.9%
2024	68,789	31,172	5,106	7,374	5,825	5,584	123,850	34.2%
2025	81,223	35,475	5,606	8,120	6,679	5,815	142,918	34.8%
**Total**	**1**,**200**,**413**	**660**,**252**	**100**,**015**	**157**,**415**	**122**,**729**	**104**,**463**	2,345,287	**31.8%**

“Share of all NEISS-coded ED visits” = target-region total ÷ all NEISS-coded visits that year. Unweighted case counts

### Distribution by body region and age group

The regional distribution of injuries varied by age group: among infants, 58.9% of cases involved the head, 27.3% the face, and 10.4% the mouth. Among toddlers, the face (40.7%) slightly exceeded the head (38.8%), with the mouth contributing 12.0%. From age 5 onward, the head share rose progressively with age, reaching 69.1% in the Older Adult group.

**Fig. 6:**
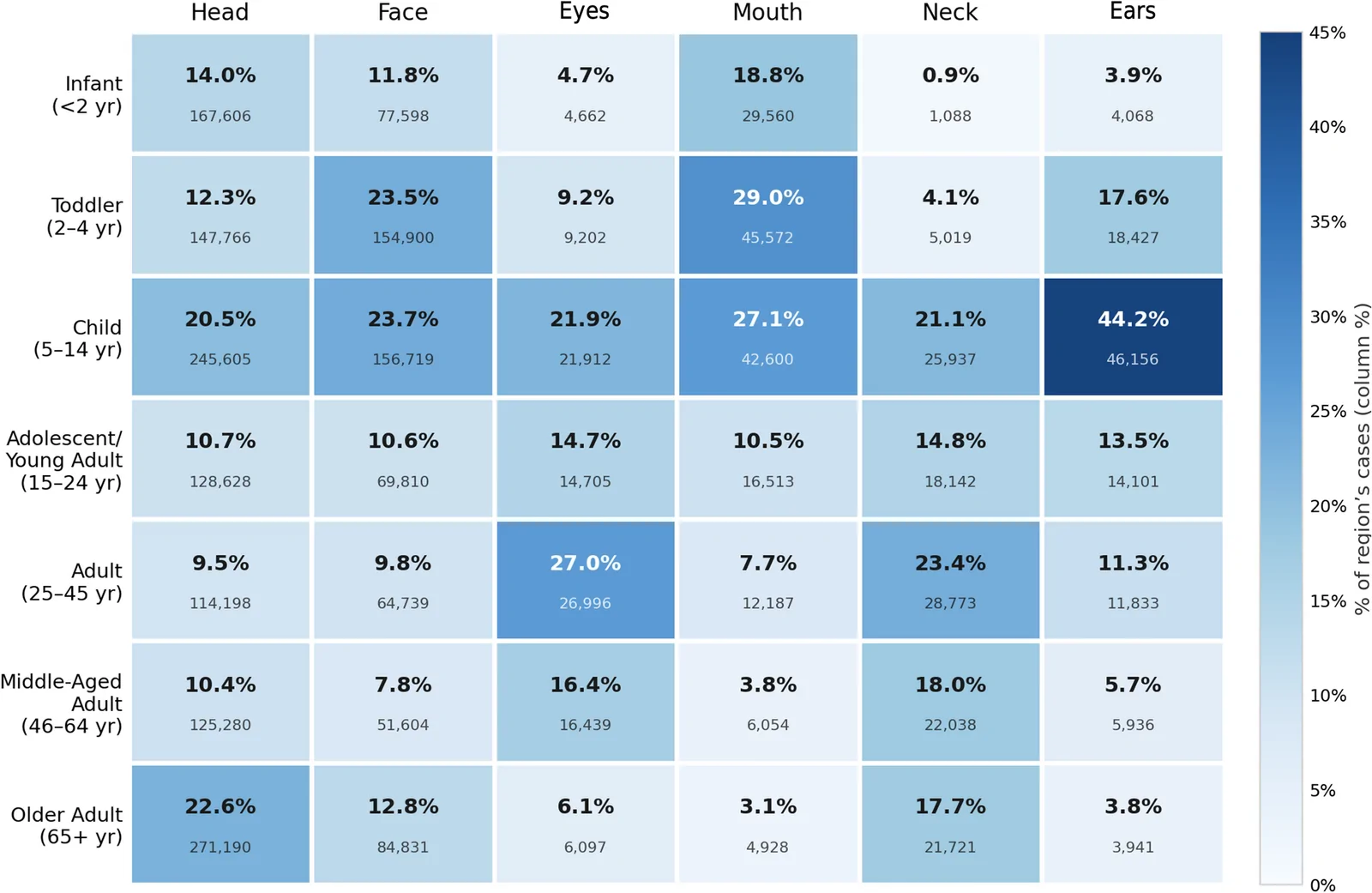
Age distribution of injuries within each anatomical body region. The heatmap shows the percentage of each region’s cases occurring within each age group, with corresponding unweighted case counts displayed below the percentages. Percentages sum to 100% within each body-region column. Records with unknown age were excluded

Each region showed a distinct age profile: 74.8% of all mouth injuries occurred in patients younger than 15 years, and 44.2% of ear injuries in children aged 5–14 years, whereas eye (27.0%) and neck (23.4%) injuries were most frequent among adults aged 25–45 years. Head injuries showed a bimodal distribution, with a first peak in early childhood and a second peak in the Older Adult group, which accounted for the largest share of head injuries (22.6%) (Fig. 6).

### Injury mechanisms

**Fig. 7:**
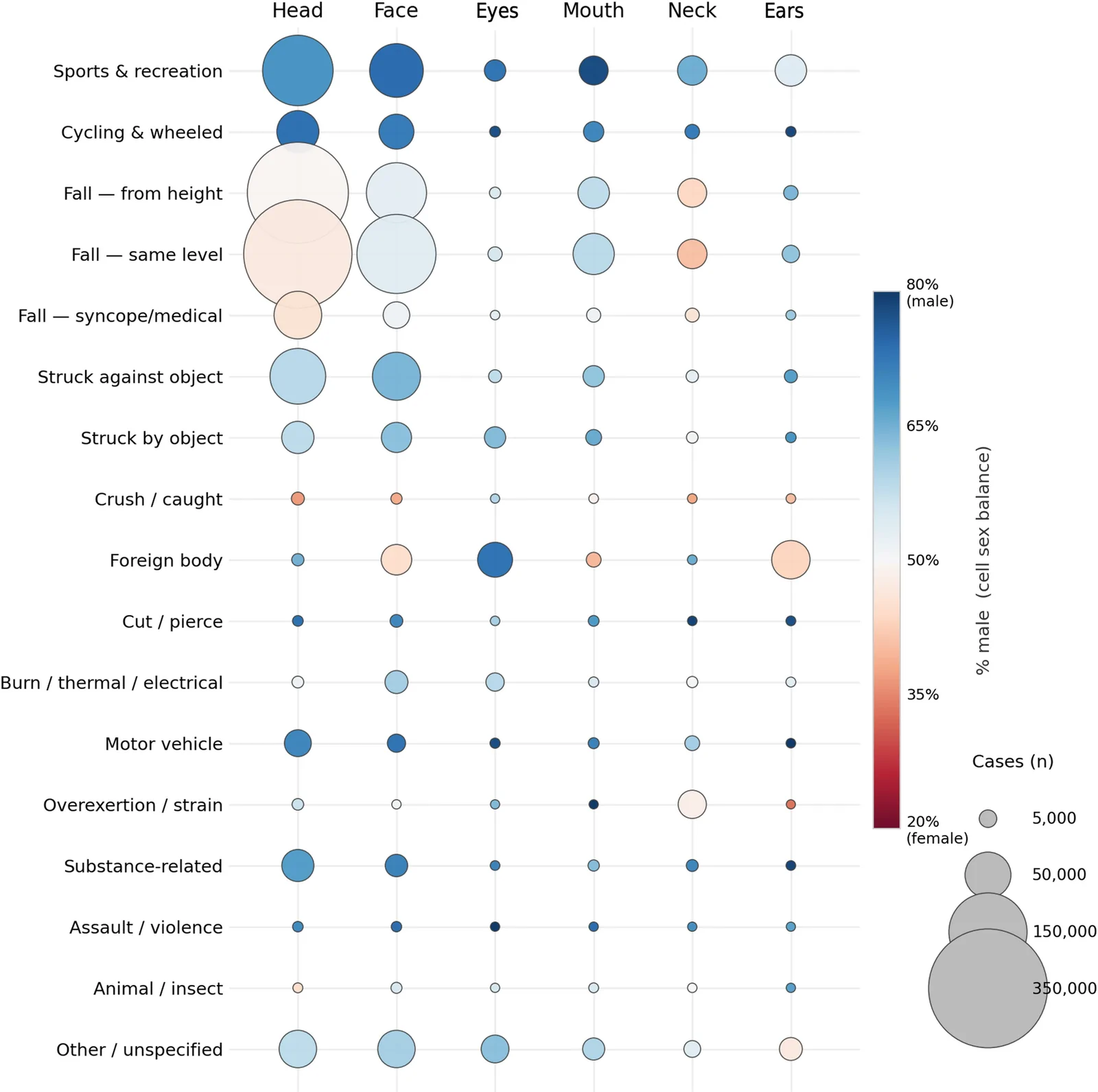
Injury mechanism by anatomical body region and patient sex. Bubble size represents the number of cases for each mechanism-region combination, while color indicates the sex distribution within that cell, ranging from male predominance (blue) through a balanced distribution (neutral) to female predominance (red). Counts are unweighted NEISS case records

Same-level falls were the most common mechanism (27.9%), followed by falls from height (21.0%), sports and recreation (14.2%), struck against an object (7.9%), foreign body inserted or aspirated (4.9%), cycling and wheeled non-motor mechanisms (4.5%), syncope-/medical-related falls (4.1%), and struck by an object (3.2%); all remaining mechanisms each accounted for 2.0% or less, and 5.9% of cases remained Other/Unspecified. Together, the three fall categories accounted for 53.0% of all cases (Table 4). Falls from height occurred predominantly at the extremes of age, with infants and toddlers accounting for 43.2% and older adults for 24.2% of these cases, and represented 41.6% of all injuries among infants. The most frequently associated products were beds (20.3%), stairs or steps (18.6%), chairs (7.2%), sofas or couches (5.3%), and table (3.5%), whereas ladders accounted for 1.8%. Same-level falls were the leading mechanism for head (30.9%), face (29.7%), and mouth (32.6%) injuries, followed in each case by falls from height. Foreign body-related mechanisms predominated among eye (36.1%) and ear (42.3%) injuries, whereas neck injuries showed a heterogeneous profile, with same-level falls (20.3%), sports and recreation (20.3%), falls from height (19.4%), and overexertion or muscle strain (18.5%) contributing similar shares. Male predominance was most pronounced for cycling and wheeled non-motor (72.6% male), assault (72.0%), cut/pierce (70.7%), motor vehicle related (70.0%), sports and recreation (69.2%), and substance-related (68.4%) mechanisms, whereas female predominance was observed for foreign-body injuries of the ear (56.7% female) and face (55.4% female), same-level falls involving the neck (59.3% female), and syncope-/medical-related falls involving the head (54.4% female) (Fig. 7 and Table 4).

**Table 4:** Cross-tabulation of injury mechanism by body region and patient sex, NEISS 2006–2025

Mechanism	Head		Face		Eyeball		Mouth		Neck		Ear		Row total
	Male	Female	Male	Female	Male	Female	Male	Female	Male	Female	Male	Female	
Fall — same level	174,392 26.7%	195,988 30.0%	104,929 16.1%	91,052 13.9%	2,069 0.3%	1,675 0.3%	29,858 4.6%	21,405 3.3%	10,166 1.6%	14,799 2.3%	4,309 0.7%	2,613 0.4%	653,255 100%
Fall — from height	**159**,**196** 32.3%	**162**,**831** 33.1%	**59**,**756** 12.1%	**52**,**653** 10.7%	**769** 0.2%	**634** 0.1%	**16**,**621** 3.4%	**12**,**143** 2.5%	**10**,**385** 2.1%	**13**,**468** 2.7%	**2**,**522** 0.5%	**1**,**406** 0.3%	**492**,**384** 100%
Sports & recreation	**106**,**328** 31.9%	**49**,**151** 14.7%	**65**,**726** 19.7%	**23**,**099** 6.9%	**8**,**639** 2.6%	**3**,**235** 1.0%	**18**,**587** 5.6%	**5**,**336** 1.6%	**16**,**236** 4.9%	**8**,**686** 2.6%	**15**,**575** 4.7%	**13**,**198** 4.0%	**333**,**796** 100%
Struck against object	**56**,**274** 30.3%	**39**,**658** 21.3%	**45**,**336** 24.4%	**25**,**416** 13.7%	**1**,**541** 0.8%	**1**,**128** 0.6%	**7**,**321** 3.9%	**4**,**413** 2.4%	**1**,**158** 0.6%	**1**,**034** 0.6%	**1**,**809** 1.0%	**895** 0.5%	**185**,**983** 100%
Foreign body — inserted/aspirated	**1**,**361** 1.2%	**738** 0.6%	**12**,**050** 10.6%	**14**,**958** 13.1%	**26**,**350** 23.1%	**9**,**716** 8.5%	**1**,**709** 1.5%	**2**,**590** 2.3%	**215** 0.2%	**114** 0.1%	**19**,**161** 16.8%	**25**,**066** 22.0%	**114**,**028** 100%
Cycling & wheeled (non-motor)	**39**,**290** 37.1%	**14**,**237** 13.4%	**25**,**991** 24.5%	**10**,**154** 9.6%	**789** 0.7%	**232** 0.2%	**7**,**337** 6.9%	**3**,**097** 2.9%	**2**,**912** 2.8%	**1**,**131** 1.1%	**552** 0.5%	**154** 0.1%	**105**,**876** 100%
Fall — syncope/medical	**31**,**742** 32.7%	**37**,**796** 38.9%	**10**,**211** 10.5%	**9**,**566** 9.8%	**108** 0.1%	**94** 0.1%	**1**,**900** 2.0%	**1**,**777** 1.8%	**1**,**626** 1.7%	**1**,**920** 2.0%	**267** 0.3%	**167** 0.2%	**97**,**174** 100%
Struck by object	**17**,**638** 23.2%	**12**,**759** 16.8%	**16**,**479** 21.6%	**9**,**744** 12.8%	**7**,**444** 9.8%	**4**,**289** 5.6%	**3**,**451** 4.5%	**1**,**816** 2.4%	**830** 1.1%	**809** 1.1%	**590** 0.8%	**272** 0.4%	**76**,**121** 100%
Substance-related	**20**,**274** 42.8%	**9**,**852** 20.8%	**9**,**491** 20.0%	**3**,**886** 8.2%	**217** 0.5%	**89** 0.2%	**878** 1.9%	**510** 1.1%	**1**,**313** 2.8%	**556** 1.2%	**251** 0.5%	**69** 0.1%	**47**,**386** 100%
Motor vehicle related	**14**,**287** 41.3%	**6**,**038** 17.5%	**5**,**736** 16.6%	**2**,**104** 6.1%	**494** 1.4%	**144** 0.4%	**833** 2.4%	**340** 1.0%	**2**,**619** 7.6%	**1**,**702** 4.9%	**224** 0.6%	**56** 0.2%	**34**,**577** 100%
Burn / thermal / electrical	**908** 3.5%	**844** 3.2%	**8**,**569** 32.6%	**5**,**532** 21.1%	**4**,**666** 17.8%	**3**,**290** 12.5%	**369** 1.4%	**301** 1.1%	**666** 2.5%	**654** 2.5%	**247** 0.9%	**216** 0.8%	**26**,**262** 100%
Overexertion / muscle strain	**927** 3.8%	**707** 2.9%	**61** 0.2%	**58** 0.2%	**33** 0.1%	**19** 0.1%	**10** 0.0%	**2** 0.0%	**10**,**854** 44.4%	**11**,**785** 48.2%	**2** 0.0%	**4** 0.0%	**24**,**462** 100%
Cut / pierce by sharp object	**709** 13.3%	**257** 4.8%	**1**,**751** 32.8%	**743** 13.9%	**136** 2.5%	**89** 1.7%	**703** 13.2%	**337** 6.3%	**178** 3.3%	**48** 0.9%	**303** 5.7%	**89** 1.7%	**5**,**343** 100%
Crush / caught	**980** 19.7%	**1**,**694** 34.1%	**505** 10.2%	**807** 16.3%	**32** 0.6%	**22** 0.4%	**153** 3.1%	**165** 3.3%	**116** 2.3%	**189** 3.8%	**123** 2.5%	**180** 3.6%	**4**,**966** 100%
Animal / insect related	**214** 8.7%	**265** 10.8%	**663** 27.0%	**544** 22.2%	**31** 1.3%	**25** 1.0%	**242** 9.9%	**198** 8.1%	**86** 3.5%	**87** 3.5%	**65** 2.7%	**32** 1.3%	**2**,**452** 100%
Assault / Interpersonal violence	**598** 30.8%	**259** 13.3%	**576** 29.7%	**205** 10.6%	**80** 4.1%	**20** 1.0%	**74** 3.8%	**26** 1.3%	**49** 2.5%	**22** 1.1%	**22** 1.1%	**11** 0.6%	**1**,**942** 100%
Other / unspecified	**24**,**400** 17.6%	**17**,**641** 12.7%	**25**,**311** 18.2%	**16**,**522** 11.9%	**13**,**739** 9.9%	**8**,**166** 5.9%	**7**,**636** 5.5%	**5**,**253** 3.8%	**3**,**370** 2.4%	**2**,**921** 2.1%	**6**,**597** 4.7%	**7**,**403** 5.3%	**138**,**959** 100%
	**649**,**518** 27.7%	**550**,**715** 23.5%	**393**,**141** 16.8%	**267**,**043** 11.4%	**67**,**137** 2.9%	**32**,**867** 1.4%	**97**,**682** 4.2%	**59**,**709** 2.5%	**62**,**779** 2.7%	**59**,**925** 2.6%	**52**,**619** 2.2%	**51**,**831** 2.2%	**2**,**344**,**966**

Cell entries are unweighted case counts, with the percentage of each mechanism row shown below the count; row percentages sum to 100% across the twelve region-by-sex columns. Records with unknown sex were excluded

### Clinical outcomes

**Fig. 8:**
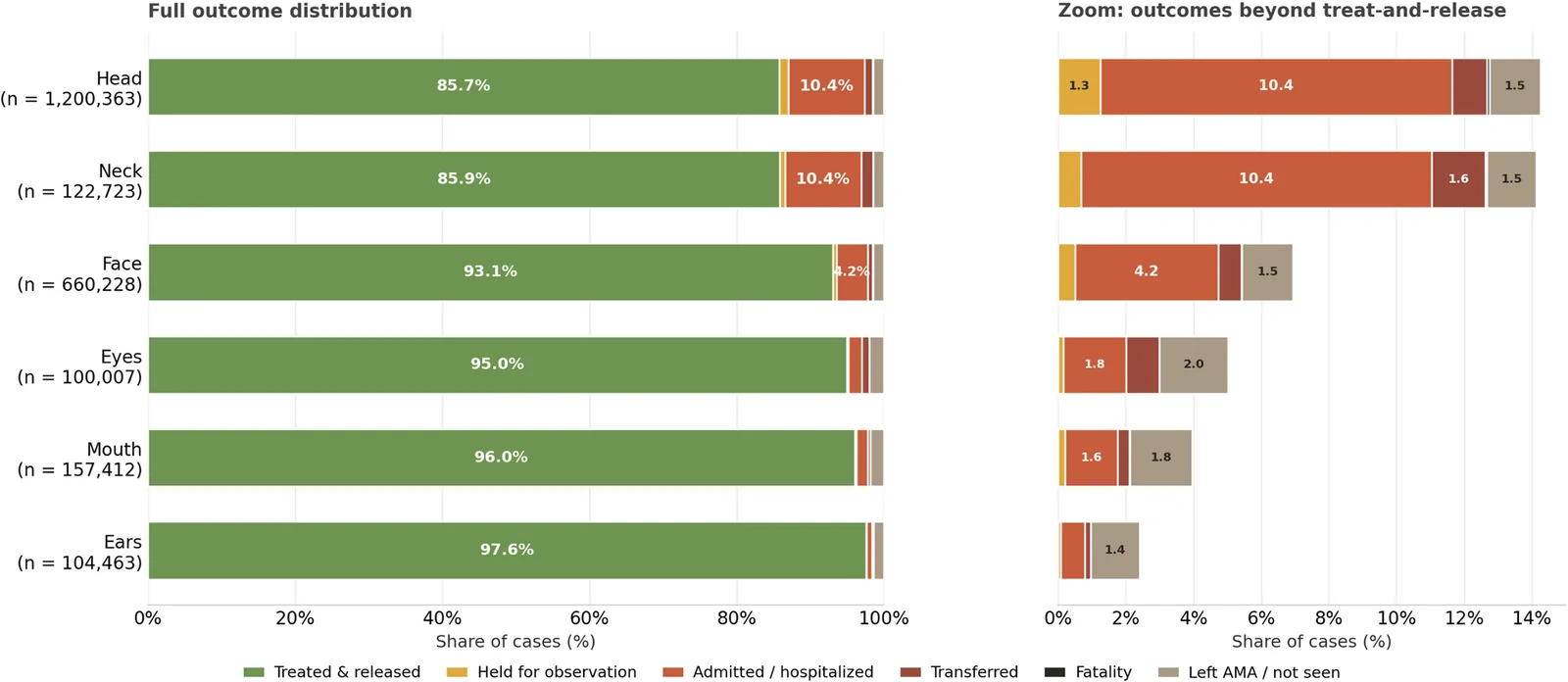
Emergency department disposition by anatomical body region. The left panel shows the full distribution of ED outcomes, including treated and released, held for observation, admitted or hospitalized, transferred, fatality, and left against medical advice or not seen. The right panel provides a magnified view of outcomes beyond treat-and-release. Percentages are calculated within each anatomical body region. AMA, against medical advice

Across the full cohort, 89.4% of patients were treated and released, 7.3% were admitted, 0.9% were transferred, 0.8% were held for observation, and 1.5% left against medical advice or were not seen; 1,062 patients (0.045%) were recorded as fatalities. The combined rate of admission, transfer, observation, and fatality, hereafter the “non-routine ED disposition” rate, was 9.0%. Female patients had a slightly higher admission (7.69% vs. 6.94%) and non-routine ED disposition rate (9.49% vs. 8.68%) than male patients, whereas case-fatality was higher among male patients (0.054% vs. 0.034%). By region, the non-routine ED disposition rate was highest for head (12.8%) and neck (12.7%) injuries, driven mainly by admissions (10.4% each), followed by face (5.4%), eye (3.0%), mouth (2.1%), and ear (1.0%) injuries (Fig. 8). Admission rates rose from 1.28% in Toddlers to 23.27% in Older Adults (18-fold) and case-fatality rates from 0.006% to 0.144% (25-fold), with a corresponding decline in the treat-and-release rate from 96.49% to 72.17%. Infants represented a partial exception, with a higher admission rate (2.61%) than Toddlers and Children (Table 5).

**Table 5:** Emergency department disposition by patient age group, NEISS 2006–2025

ED disposition	Infant	Toddler	Child	Adolescent / Young Adult	Adult	Middle-Aged Adult	Older Adult	Row total
	< 2 yr	2–4 yr	5–14 yr	15–24 yr	25–45 yr	46–64 yr	65 + yr	
Treated and released	**267**,**845** 94.12%	**367**,**520** 96.49%	**515**,**991** 95.75%	**244**,**661** 93.42%	**231**,**895** 89.64%	**185**,**758** 81.71%	**283**,**404** 72.17%	**2**,**097**,**074** 89.43%
Admitted to index hospital	**7**,**434** 2.61%	**4**,**887** 1.28%	**11**,**154** 2.07%	**8**,**919** 3.41%	**15**,**530** 6.00%	**30**,**976** 13.63%	**91**,**360** 23.27%	**170**,**260** 7.26%
Transferred to another facility	**1**,**890** 0.66%	**1**,**198** 0.31%	**2**,**564** 0.48%	**1**,**944** 0.74%	**2**,**601** 1.01%	**3**,**089** 1.36%	**7**,**394** 1.88%	**20**,**680** 0.88%
Held for observation	**1**,**993** 0.70%	**1**,**345** 0.35%	**2**,**688** 0.50%	**1**,**572** 0.60%	**1**,**805** 0.70%	**3**,**006** 1.32%	**7**,**316** 1.86%	**19**,**725** 0.84%
Left AMA / not seen	**5**,**384** 1.89%	**5**,**908** 1.55%	**6**,**463** 1.20%	**4**,**727** 1.80%	**6**,**753** 2.61%	**4**,**316** 1.90%	**2**,**648** 0.67%	**36**,**199** 1.54%
Fatality	**29** 0.010%	**22** 0.006%	**58** 0.011%	**63** 0.024%	**117** 0.045%	**198** 0.087%	**566** 0.144%	**1**,**053** 0.045%
Column total	**284**,**575** 100%	**380**,**880** 100%	**538**,**918** 100%	**261**,**886** 100%	**258**,**701** 100%	**227**,**343** 100%	**392**,**688** 100%	**2**,**344**,**991**

Cells show unweighted case counts with the percentage within each age group below the count; column percentages sum to 100%. The row-total column shows the overall count and percentage for each ED disposition category across included records. Records with unknown age and/or unrecorded ED disposition were excluded. AMA, against medical advice

The non-routine ED disposition rate also varied by mechanism. Among mechanisms with at least 1,000 cases, it was highest for syncope-/medical-related falls (33.9%; case-fatality 0.223%), followed by substance-related cases (23.6%; 0.063%), motor vehicle related cases (20.7%; 0.309%), and burn/thermal/electrical cases (18.7%; 0.114%), compared with 10.9% for falls from height and 9.9% for same-level falls. Foreign-body, overexertion, struck-against-object, struck-by-object, and sports-related injuries had the lowest rates (1.2–3.1%). The three fall categories accounted for 66.9% of all fatalities (270 falls from height, 224 same-level falls, and 217 syncope-/medical-related falls), and 899 of the 1,062 deaths (84.7%) involved the head.

### Injury setting and associated products/surfaces

**Fig. 9:**
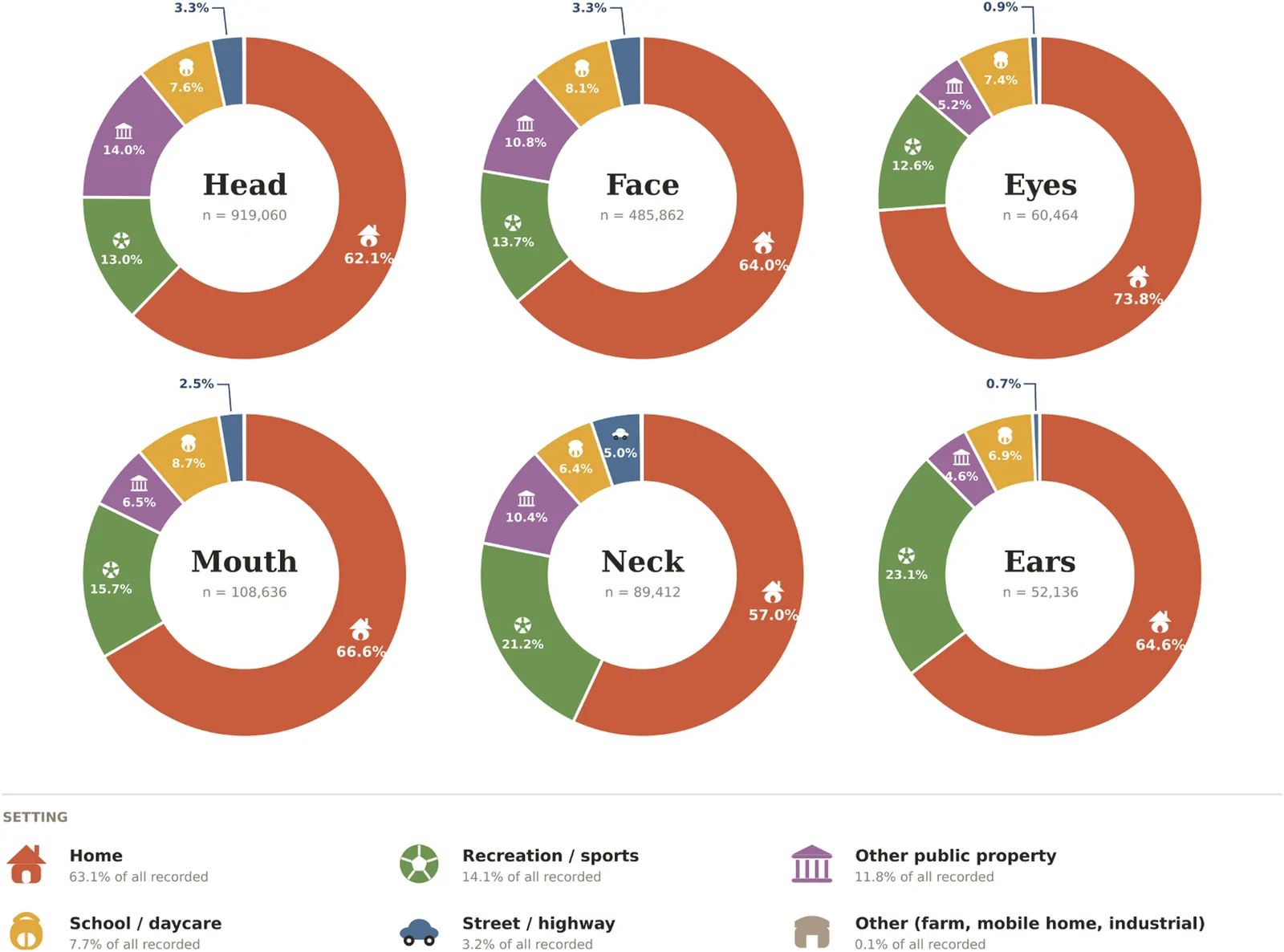
Injury setting by anatomical body region among records with a documented injury location. Donut charts show the proportional distribution of injury settings for each body region, including home, recreation or sports, school or daycare, street or highway, other public property, and other settings. Percentages are calculated among records with a recorded location; unrecorded locations were excluded

Injury location was documented for 73.1% of records. Among these, the home was the most common setting (63.1%), ranging from 57.0% for neck to 73.8% for eye injuries. Places of recreation or sports accounted for 14.1% of cases, with the highest shares for ear (23.1%) and neck (21.2%) injuries, followed by other public property (11.8%), school or daycare (7.7%), and street or highway (3.2%) (Fig. 9).

**Fig. 10:**
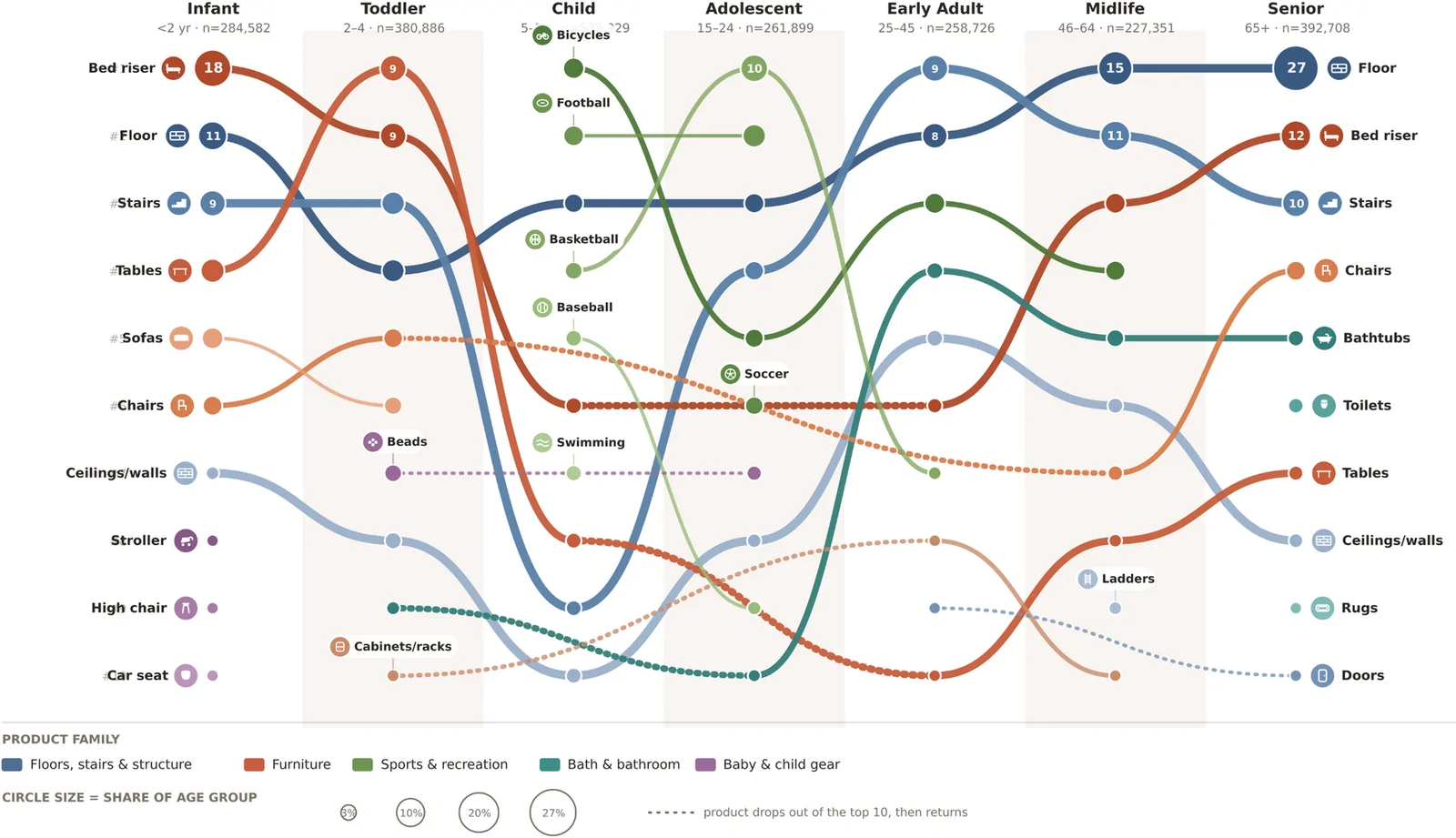
Most frequently coded associated products or environmental surfaces by age group. The figure displays the most frequently coded associated products or environmental surfaces recorded for target-region injuries across age groups. Circle size corresponds to the product’s share within each age group, and line color indicates broader product family. Solid lines indicate products remaining among the top categories across adjacent age groups, whereas dotted lines indicate products that drop out of the top ten and later reappear. Counts are unweighted NEISS case records

Associated products and surfaces differed by age group, although floors and stairs ranked among the ten most frequent in every age group. Beds were the most frequent among infants (17.8%), followed by floors (10.6%) and stairs (8.6%), and tables among toddlers (8.9%), followed by beds (8.7%) and stairs (6.8%). Sports-related products, including bicycles, football, basketball, baseball, soccer, and swimming-related products, were most prominent among Children and Adolescents/Young Adults; in older age groups, only bicycles and basketball remained among the ten most frequent. Among Older Adults, floors were most frequent (26.6%), followed by beds (11.7%), stairs (9.5%), and bathtubs (3.3%) (Fig. 10).

## Discussion

This study provides a large-scale, two-decade overview of head, face, and neck injuries captured in NEISS-participating U.S. emergency departments, integrating demographic patterns, anatomical distribution, injury mechanisms, clinical disposition, settings, and associated products or surfaces. The findings demonstrate that these injuries accounted for nearly one-third of all NEISS-coded ED visits, with the head representing the most frequently injured region and the main driver of the observed increase in annual case volume. Falls were the dominant mechanism, although injury mechanisms, anatomical patterns, and associated products or environmental surfaces varied substantially by age group, sex, and body region. The non-routine ED disposition rate increased sharply with advancing age and was particularly high after syncope- or medical-related falls, motor vehicle-related injuries, substance-related injuries, and burn, thermal, or electrical mechanisms. Finally, the home was the most common injury setting, thereby alluding that prevention strategies may need to be specific to age, mechanism, and environment. The automated narrative classifier further enabled mechanism-level analysis across the full cohort and reduced the residual “Other/Unspecified” category to 5.9%, supporting a more granular interpretation of injury circumstances.

Over time, injuries involving the face, head, neck, mouth, ears, and eyes have comprised an increasing proportion of NEISS-coded emergency department presentations. Between 2006 and 2025, the total observed annual case volume increased by 35.2%, while the proportion of all NEISS-coded ED visits attributable to these anatomical regions rose from 29.1% to 34.8%. This increase was driven predominantly by head injuries, whereas annual counts for face, eye, and mouth injuries remained comparatively stable. These findings are consistent with prior reports describing increasing ED visits for head and facial trauma [7, 8, 12]. Similar increases in fall-related injury hospitalizations have been reported among older adults, including disproportionate increases in facial fracture and head injury hospitalizations [9, 10]. Several factors may explain the increase in head injuries. Population aging may contribute, as older adults are more susceptible to fall-related head trauma and more likely to require ED evaluation after minor trauma. In addition, increased clinical awareness of traumatic brain injury, lower thresholds for imaging and observation, and greater anticoagulant and antiplatelet use among older adults may have increased ED presentation and evaluation rates for head injuries [11, 12]. The decrease in case volume observed in 2020 across all anatomical regions is also consistent with the documented reduction in ED utilization during the COVID-19 pandemic, including marked declines in injury-related ED visits [24, 25]. Notably, although the absolute 2020 case volume was the lowest of the entire study period and 13.5% below the 2019 level, the proportion of all NEISS-coded ED visits attributable to the target regions remained virtually the same, with 32.8% vs. 32.9% in 2019 and 2020, respectively, indicating that the pandemic-related decline affected target-region injuries to a similar degree as other NEISS-coded injuries.

Overall, the cohort showed a male predominance and a young median age, consistent with prior ED- and trauma-based studies of head and neck injuries, facial injuries, and craniofacial soft tissue trauma [1, 17, 26]. However, the present study expands on these observations by demonstrating that sex distribution varied substantially by age group, anatomical region, and injury mechanism. Male predominance was most pronounced for eye and mouth injuries and for sports, cycling and wheeled non-motor, motor vehicle-related, cut or pierce, substance-related, and assault-related mechanisms. These patterns may reflect greater exposure to contact sports, cycling, occupational hazards, high-energy mechanisms, and risk-prone recreational or interpersonal activities among boys and men [1, 7, 27]. In contrast, female patients exceeded male patients in the older adult group, a finding that likely reflects greater longevity among women, higher prevalence of osteoporosis and frailty, and the higher burden of fall-related injury among elderly women [1, 28]. Similarly, the female predominance observed in same-level neck falls and syncope- or medical-related head falls is consistent with the higher fall burden among older women [28]. In contrast, the female predominance in ear foreign-body injuries may reflect jewelry-related exposures, as previously described in pediatric foreign-body studies [13]. Anatomically, head injuries showed a bimodal pattern, with peaks in infants, toddlers and children and a second peak among older adults. In young children, this likely reflects developmental vulnerability, including a relatively large head-to-body ratio, immature protective reflexes, developing motor coordination, and frequent falls from furniture or standing height [17, 29]. The concentration of mouth injuries in children younger than 15 years is consistent with the epidemiology of dental trauma, which often peaks during early childhood and primary dentition [30, 31]. Ear injuries clustered in school-aged children, consistent with the known burden of pediatric ear foreign bodies, while eye injuries peaked among adults aged 25 to 45 years, reflecting patterns previously described for ocular trauma [4, 13]. 

Falls represented the dominant mechanism of injury in this cohort, accounting for 53.0% of all cases when all fall subcategories were combined. This finding is consistent with prior national, institutional, and global analyses identifying falls as a leading mechanism of head, neck, facial, and craniomaxillofacial trauma [1, 6, 32, 33]. Importantly, the present study provides additional granularity by distinguishing between same-level falls, falls from height, and syncope- or medical-related falls and by demonstrating mechanism-specific differences across anatomical regions. Of note, falls from height in this analysis predominantly represented low-level falls from beds, furniture, and stairs which needs to be kept in mind when comparing them with high-energy falls from height reported in other trauma center-based studies. Same-level falls were the leading mechanism for head, face, and mouth injuries, whereas foreign body-related mechanisms predominated among eye and ear injuries. Neck injuries showed a more heterogeneous mechanism profile, with same-level falls, sports and recreation, falls from height, and overexertion or muscle strain contributing similar proportions. The diverse mechanism profile of neck injuries likely reflects the anatomical and functional complexity of the cervical region, which is vulnerable to direct impact, acceleration-deceleration forces, sports-related loading, and muscular strain. Head and face injuries commonly occur during direct impact after falls, particularly at the extremes of age. Eye and ear injuries, in contrast, are more strongly influenced by foreign body exposure, manual exploration, occupational or household debris, and child-specific behaviors.

Although most patients were treated and released from the ED, non-routine ED dispositions varied substantially by age, anatomical region, and mechanism. The high overall treat-and-release proportion is consistent with prior work reporting that most head and neck injury-related ED visits do not result in hospitalization [1]. Generally, the young median age and high “treat-and-release” proportion of the analyzed cohort should be interpreted in light of the sampling frame applied in NEISS, which includes EDs of all sizes and a separate children’s hospital stratum and is restricted to injuries associated with consumer products. The present findings may therefore not be directly comparable with conventional trauma center- or trauma registry-based cohorts. However, the present study demonstrates a pronounced age gradient: admission, non-routine ED disposition, and case-fatality rates increased markedly from toddlers to older adults. This pattern is consistent with the broader injury literature showing that older adults account for a disproportionate share of injury-related ED visits and hospitalizations, particularly for falls [10, 34]. Several mechanisms may explain this gradient. Older adults are more likely to have frailty, comorbid disease, impaired balance, polypharmacy, anticoagulant use, and reduced physiological reserve, all of which can increase the likelihood of admission or death after head, face, or neck trauma [28, 35]. It ought to be noted that these factors are however not recorded in NEISS and hence could not be included in the analysis of the present study. Mechanism-specific outcomes are consistent with this interpretation. Syncope- or medical-related falls had the highest non-routine ED disposition rate, suggesting that underlying medical conditions such as arrhythmias, orthostatic hypotension, medication effects, dehydration, or neurologic events may contribute to both injury occurrence and subsequent clinical severity. Motor vehicle-related injuries were uncommon but had the highest case-fatality rate, consistent with the greater kinetic energy transfer associated with these events. Substance-related cases and burn, thermal, or electrical injuries also showed elevated non-routine ED disposition rates.

The analysis of injury setting and associated products or environmental surfaces highlights the importance of environmental context. Among cases with a recorded location, the home was the most common injury setting across all anatomical regions. This finding aligns with prior NEISS-based analyses showing that at-home injuries account for a substantial proportion of facial injuries and that the proportion of home injuries increases with age [17]. Product profiles varied strongly by age group. Beds were the most frequently coded associated product/surface among infants, tables among toddlers, sports-related products among children and adolescents, and floors among older adults. These findings mirror prior product-focused NEISS analyses, which have shown that home furnishings and fixtures are major contributors to injuries in infants and young children, while sports and recreational products become more prominent in older children and adolescents [16, 36, 37]. 

Taken together, these findings could have several clinical and public health implications, which are summarized by age group in Table 6. First, the concentration of injuries in the home setting suggests that prevention efforts may benefit from emphasizing age-specific environmental modification, including safe sleep and furniture practices for infants, supervision and furniture safety for toddlers, and fall-hazard reduction for older adults. Second, the dominance of falls and the steep age gradient in non-routine ED disposition align with current recommendations for routine fall-risk assessment, medication review, vision assessment, balance and strength interventions, treatment of sarcopenia and home-safety counseling for older adults, particularly those presenting with head or neck injuries [28, 35]. Third, the higher non-routine ED disposition rates observed in older adults, patients with syncope- or medical-related falls, motor vehicle-related injuries, burns, and substance-related presentations may warrant particular attention during ED evaluation, although post-ED disposition should be seen as only an indirect surrogate of clinical severity. Fourth, product- and activity-specific counseling may be useful across pediatric and adolescent populations, including education regarding furniture hazards, toy-related injuries, sports-related dental trauma, cycling injuries, and appropriate protective equipment use [14, 16, 27, 36, 37]. Rather than supporting a single uniform prevention strategy, the present findings suggest that effective prevention may need to be age-, mechanism-, and setting-specific.

**Table 6:** Age-specific injury patterns and potential prevention focus areas, NEISS 2006–2025

Age group	Main regions	Leading mechanisms	Main setting*	Leading products/surfaces	Admission	Potential prevention focus
Infant (< 2 y)	Head 58.9%, face 27.3%	Fall from height 41.6%, same-level fall 35.2%	Home 89.6%	Beds 17.8%, floors 10.6%, stairs 8.6%	2.6%	Safe sleep surfaces, no unattended placement on elevated surfaces, stair gates
Toddler (2–4 y)	Face 40.7%, head 38.8%	Same-level fall 33.2%, fall from height 24.8%	Home 79.5%	Table 8.9%, beds 8.7%, stairs 6.8%	1.3%	Furniture safety, stair gates, supervision
Child (5–14 y)	Head 45.6%, face 29.1%	Sports/recreation 30.1%, same-level fall 18.2%	Home 44.3%, recreation 27.0%	Bicycles 5.7%, football 5.4%, basketball 3.9%	2.1%	Helmets, mouthguards, playground safety
Adolescent/Young Adult (15–24 y)	Head 49.1%, face 26.7%	Sports/recreation 38.4%, same-level fall 11.3%	Recreation 39.2%, home 33.1%	Basketball 9.7%, football 6.9%, floors 5.1%	3.4%	Sport-specific protective equipment
Adult (25–45 y)	Head 44.1%, face 25.0%	Same-level fall 18.2%, fall from height 13.0%	Home 61.1%	Stairs 9.0%, floors 8.3%, bicycles 5.5%	6.0%	Eye protection, ladder and stair safety, cycling helmets
Middle-Aged Adult (46–64 y)	Head 55.1%, face 22.7%	Same-level fall 28.8%, fall from height 20.1%	Home 69.9%	Floors 14.8%, stairs 11.3%, beds 5.4%	13.6%	Home fall-hazard reduction, evaluation of medical causes of falls
Older Adult (≥ 65 y)	Head 69.1%, face 21.6%	Same-level fall 47.5%, fall from height 30.3%	Home 69.3%	Floors 26.6%, beds 11.7%, stairs 9.5%	23.3%	Multifactorial fall-risk assessment, medication review, home-safety modification

Unweighted case records; percentages within each age group. *Among records with a documented location

### Limitations

This study has several limitations. First, as a retrospective analysis of the NEISS database, it is subject to the inherent constraints of administrative surveillance data, including potential coding errors, variability in abstractor interpretation, and limited clinical detail. Second, NEISS captures only injuries presenting to participating EDs and does not include injuries managed in outpatient clinics, urgent care settings, dental offices, or those not receiving medical attention. The true burden of less severe injuries is therefore likely underestimated. Third, NEISS was specifically designed to capture consumer-product-related injuries, so that injuries without an associated product are not represented on a systematic level. Fourth, the present analysis used unweighted case records rather than NEISS sampling weights. Consequently, the counts and proportions reported here reflect the observed NEISS sample and should not be interpreted as national population estimates. Since NEISS hospitals are sampled with unequal selection probabilities across strata, including a separate children’s hospital stratum, the unweighted sample may over-represent pediatric presentations, which may have influenced the reported age distribution and injury mechanisms. Fifth, NEISS does not contain detailed clinical variables such as imaging findings, injury severity scores, operative interventions, comorbidities, anticoagulant use, or long-term functional outcomes. ED disposition was therefore used as a pragmatic proxy for short-term clinical acuity, but it cannot fully capture injury complexity or downstream morbidity. Because NEISS records a primary coded body part and limited clinical detail, we could not determine whether admissions, transfers, or observations were attributable solely to the indexed head, face, or neck injury or to concomitant injuries and comorbid conditions. Sixth, injury mechanisms were derived from brief free-text narratives using an automated rule-based classifier. Although the classifier was developed iteratively and validated using internal procedures, misclassification remains possible, particularly for ambiguous or abbreviation-heavy narratives. Seventh, the 20-year study period spans changes in clinical practice, ED utilization, coding conventions, product exposures, and public health conditions, including the COVID-19 pandemic, all of which may have influenced temporal trends independently of true changes in injury occurrence. Eighth, the analyses were purely descriptive, and the reported associations between patient characteristics, injury mechanisms, and outcomes should, due to the inherent limitations of the utilized database, not be interpreted as causal. Despite these limitations, the large sample size, standardized data structure, long observation period, and integrated analysis across anatomical regions, mechanisms, age groups, and associated products or environmental surfaces provide a comprehensive descriptive overview of head, face, and neck injuries captured in NEISS.

## Conclusions

This 20-year NEISS analysis demonstrates that injuries to the head, face, neck, eyes, ears, and mouth represent a substantial and increasing share of NEISS-coded ED presentations, with the observed increase driven primarily by head injuries. Falls were the dominant mechanism, non-routine ED disposition increased sharply with age, and injury patterns varied substantially by anatomical region, sex, mechanism, setting, and associated products or surfaces. 

## Data Availability

The datasets used and/or analysed during the current study are available from the corresponding author on reasonable request.

## References

[1] Sethi RK, et al. Epidemiological survey of head and neck injuries and trauma in the United States. Otolaryngol Head Neck Surg. 2014;151(5):776–84. 10.1177/0194599814546112. PMC4481864 25139950

[2] Peterson C, et al. Economic Cost of Injury - United States, 2019. MMWR Morb Mortal Wkly Rep. 2021;70(48):1655–9. 10.15585/mmwr.mm7048a1. PMC8641568 34855726

[3] Allareddy V, Allareddy V, Nalliah RP. Epidemiology of facial fracture injuries. J Oral Maxillofac Surg. 2011;69(10):2613–8. 10.1016/j.joms.2011.02.057. 21683499

[4] Ramirez DA, et al. Ocular Injury in United States Emergency Departments: Seasonality and Annual Trends Estimated from a Nationally Representative Dataset. Am J Ophthalmol. 2018;191:149–55. 10.1016/j.ajo.2018.04.020. PMC6014923 29750945

[5] Iftikhar M, et al. Epidemiology of ophthalmic trauma in the United States from 2009–2018: A Nationwide Emergency Department Sample Analysis. Injury. 2024;55(3):111209. 10.1016/j.injury.2023.111209. 38012902

[6] Lalloo R, et al. Epidemiology of facial fractures: incidence, prevalence and years lived with disability estimates from the Global Burden of Disease 2017 study. Inj Prev. 2020;26(Supp 1):i27–35. 10.1136/injuryprev-2019-043297. PMC7571355 31915268

[7] Onor GI Jr., et al. Epidemiological trends of facial fractures in the United States. Am J Emerg Med. 2025;96:176–9. 10.1016/j.ajem.2025.06.040. 40582102

[8] Han MD, et al. Epidemiology of Facial Fractures Encountered in the Emergency Department Setting in the United States: An Update of Nationwide Estimates. J Oral Maxillofac Surg. 2026;84(6):887–95. 10.1016/j.joms.2026.01.015. 41688037

[9] Aminian P, et al. Hospitalisation trends for falls and facial fractures in elderly Australian: A national ecological analysis, from 2013-14 to 2022-23. Arch Gerontol Geriatr. 2026;143:106147. 10.1016/j.archger.2026.106147. 41643297

[10] Orces CH. Trends in Hospitalization for Fall-Related Injuries in US Older Adults, 2001–2021. ScientificWorldJournal. 2025;2025:8340466. 10.1155/tswj/8340466. PMC12033062 40292182

[11] DiMaggio CJ, et al. The Epidemiology of Emergency Department Trauma Discharges in the United States. Acad Emerg Med. 2017;24(10):1244–56. 10.1111/acem.13223. PMC5647215 28493608

[12] Gaw CE, Zonfrillo MR. Emergency department visits for head trauma in the United States. BMC Emerg Med. 2016;16:5. 10.1186/s12873-016-0071-8. PMC4717651 26781953

[13] Xiao CC, Kshirsagar RS, Rivero A. Pediatric foreign bodies of the ear: A 10-year national analysis. Int J Pediatr Otorhinolaryngol. 2020;138:110354. 10.1016/j.ijporl.2020.110354. 33152957

[14] Montero E, et al. Pediatric Sports- and Recreation-Related Dental Injuries Treated in US Emergency Departments. Clin Pediatr (Phila). 2019;58(11–12):1262–70. 10.1177/0009922819853768. 31179757

[15] Lin JS, et al. Factors Associated With Head and Neck Polytrauma Presentation and Admissions at Emergency Departments of Varying Sizes. J Craniofac Surg. 2025;36(1):41–7. 10.1097/scs.0000000000010371. 38830051

[16] Ali B, et al. Products and activities associated with non-fatal traumatic brain injuries in children and adolescents - United States 2010–2013. Brain Inj. 2019;33(11):1425–9. 10.1080/02699052.2019.1631483. 31355679

[17] Kim EJ, Bustos VP, Lee BT. Sources of Facial Injury Across Age Groups: A Nationwide Overview Using the National Electronic Injury Surveillance System Database. J Craniofac Surg. 2023;34(7):1927–30. 10.1097/scs.0000000000009582. 37582255

[18] Kamboj AK, et al. Nonfatal Pediatric Injuries Associated With Consumer Products and Sports and Recreational Activities in the United States. Pediatr Emerg Care. 2020;36(9):e485–93. 10.1097/pec.0000000000001778. 30829840

[19] Povolotskiy R, et al. Head and Neck Injuries Associated With Cell Phone Use. JAMA Otolaryngology–Head Neck Surg. 2020;146(2):122–7. 10.1001/jamaoto.2019.3678. PMC6902153 31804678

[20] Schroeder T. A.K., The NEISS Sample (Design and Implementation) 1997 to Present. U.S. Consumer Product Safety Commission; 2001.

[21] U.S. Consumer Product Safety Commission. National Electronic Injury Surveillance System (NEISS). [cited 2026 June]; Available from: https://www.cpsc.gov/Research--Statistics/NEISS-Injury-Data.

[22] National Center for Health Statistics. External cause-of-injury matrices. [cited 2026 June]; Available from: https://www.cdc.gov/nchs/injury/.

[23] Proposed framework for presenting injury data using ICD-10-CM external cause of injury codes. 2014: Atlanta, GA.

[24] Law RK, et al. Injury-Related Emergency Department Visits During the COVID-19 Pandemic. Am J Prev Med. 2022;63(1):43–50. 10.1016/j.amepre.2022.01.018. PMC8858709 35292198

[25] Hartnett KP, et al. Impact of the COVID-19 Pandemic on Emergency Department Visits - United States, January 1, 2019-May 30, 2020. MMWR Morb Mortal Wkly Rep. 2020;69(23):699–704. 10.15585/mmwr.mm6923e1. PMC7315789 32525856

[26] Yin AA, et al. Epidemiological and clinical characteristics of craniofacial soft tissue injuries in a plastic emergency department in Xi’an, China: a retrospective study of 22887 patients from 2019 to 2023. BMC Public Health. 2025;25(1):854. 10.1186/s12889-025-22068-w. PMC11877798 40033249

[27] Clementi EA, et al. Age- and Sex-Related Trends in Bicycle-Related Craniomaxillofacial Injuries: A 10-Year Analysis of National Emergency Department Data. J Craniofac Surg. 2026;37(3–4):474–8. 10.1097/scs.0000000000011816. 40773364

[28] Colón-Emeric CS, et al. Risk Assessment and Prevention of Falls in Older Community-Dwelling Adults: A Review. JAMA. 2024;331(16):1397–406. 10.1001/jama.2024.1416. PMC12224174 38536167

[29] Gassner R, et al. Craniomaxillofacial trauma in children: a review of 3,385 cases with 6,060 injuries in 10 years. J Oral Maxillofac Surg. 2004;62(4):399–407. 10.1016/j.joms.2003.05.013. 15085503

[30] Wilson S, et al. Epidemiology of dental trauma treated in an urban pediatric emergency department. Pediatr Emerg Care. 1997;13(1):12–5. 10.1097/00006565-199702000-00004. 9061727

[31] Zhang JL, et al. A two-year retrospective study on traumatic dental injury in the primary dentition. Med (Baltim). 2023;102(45):e35750. 10.1097/md.0000000000035750. PMC10637491 37960738

[32] Long S, et al. Patterns of Facial Fractures in a Major Metropolitan Level 1 Trauma Center: A 10-year Experience. Laryngoscope. 2021;131(7):E2176–80. 10.1002/lary.29397. 33433012

[33] Rangarajan S, et al. Epidemiology of Craniomaxillofacial Fractures Over a 5-year Period at a Midwestern Level 1 Trauma Center Serving a Large Rural Population. J Oral Maxillofac Surg. 2024;82(6):663–70. 10.1016/j.joms.2024.03.002. 38527728

[34] Moreland B, Lee R. Emergency Department Visits and Hospitalizations for Selected Nonfatal Injuries Among Adults Aged ≥ 65 Years - United States, 2018. MMWR Morb Mortal Wkly Rep. 2021;70(18):661–6. 10.15585/mmwr.mm7018a1. PMC9368752 33956783

[35] Ganz DA, Latham NK. Prevention of Falls in Community-Dwelling Older Adults. N Engl J Med. 2020;382(8):734–43. 10.1056/NEJMcp1903252. 32074420

[36] Gursky AK, et al. Exploring trends in pediatric craniofacial furniture accidents: Implications for prevention, safety counseling, and future advocacy initiatives. Injury. 2025;56(7):112373. 10.1016/j.injury.2025.112373. 40347808

[37] Treger D, Plotsker E, Thaller SR. A Characterization of Pediatric Craniofacial Injuries from Children’s Toys. J Craniofac Surg. 2023;34(4):1256–8. 10.1097/scs.0000000000009322. 37101322

